# Therapeutic targets for age-related macular degeneration: proteome-wide Mendelian randomization and colocalization analyses

**DOI:** 10.3389/fneur.2024.1400557

**Published:** 2024-06-06

**Authors:** Kun-Lin Pu, Hong Kang, Li Li

**Affiliations:** ^1^Pengzhou Hospital of Traditional Chinese Medicine, Chengdu, China; ^2^Department of Thoracic Surgery, Sichuan Cancer Hospital, Chengdu, China

**Keywords:** Mendelian randomization, protein, therapeutic target, age-related macular degeneration, drug prediction

## Abstract

**Background:**

Currently, effective therapeutic drugs for age-related macular degeneration (AMD) are urgently needed, and it is crucial to explore new treatment targets. The proteome is indispensable for exploring disease targets, so we conducted a Mendelian randomization (MR) of the proteome to identify new targets for AMD and its related subtypes.

**Methods:**

The plasma protein level data used in this study were obtained from two large-scale studies of protein quantitative trait loci (pQTL), comprising 35,559 and 54,219 samples, respectively. The expression quantitative trait loci (eQTL) data were sourced from eQTLGen and GTEx Version 8. The discovery set for AMD data and subtypes was derived from the FinnGen study, consisting of 9,721 AMD cases and 381,339 controls, 5,239 wet AMD cases and 273,920 controls, and 6,651 dry AMD cases and 272,504 controls. The replication set for AMD data was obtained from the study by Winkler TW et al., comprising 14,034 cases and 91,234 controls. Summary Mendelian randomization (SMR) analysis was employed to assess the association between QTL data and AMD and its subtypes, while colocalization analysis was performed to determine whether they share causal variants. Additionally, chemical exploration and molecular docking were utilized to validate potential drugs targeting the identified proteins.

**Results:**

SMR and colocalization analysis jointly identified risk-associated proteins for AMD and its subtypes, including 5 proteins (WARS1, BRD2, IL20RB, TGFB1, TNFRSF10A) associated with AMD, 2 proteins (WARS1, IL20RB) associated with Dry-AMD, and 9 proteins (COL10A1, WARS1, VTN, SDF2, LBP, CD226, TGFB1, TNFRSF10A, CSF2) associated with Wet-AMD. The results revealed potential therapeutic chemicals, and molecular docking indicated a good binding between the chemicals and protein structures.

**Conclusion:**

Proteome-wide MR have identified risk-associated proteins for AMD and its subtypes, suggesting that these proteins may serve as potential therapeutic targets worthy of further clinical investigation.

## 1 Introduction

Age-related macular degeneration (AMD) is a leading cause of blindness in adults aged 60 and above worldwide, characterized by progressive degeneration of the retinal pigment epithelium, retina, and choroidal capillaries (1, 2). The global prevalence of AMD is approximately 8.7%, affecting over 190 million people (3). The global cost of vision loss due to AMD is estimated to exceed 300 billion U.S. dollars, and this figure is expected to continue rising (1), imposing a significant burden on society as a whole.

AMD can be divided into two types: neovascular (wet) AMD and non-neovascular (dry) AMD, with approximately 80% classified as dry AMD and the remaining 20% as wet AMD (4). Dry AMD, also known as geographic atrophy, typically has a better visual prognosis compared to wet AMD, which accounts for about 80% of severe vision loss in AMD cases. Age, smoking, body mass index, hypertension, hyperlipidemia, and genetics have been identified as important risk factors for AMD (5), but the exact pathogenesis of the disease remains unclear. Currently, all clinically approved treatments for AMD cannot cure the condition, and therapy is primarily based on the use of anti-vascular endothelial growth factor drugs (6). Therefore, research focused on identifying therapeutic targets for AMD is crucial to develop effective treatments. Proteins, due to their specific binding sites or regions, can often serve as targeted binding sites for small molecules or biologics, allowing the precise and controlled development of drugs that interact with proteins (7). The advancement of proteomic technologies has led to an increasing number of studies exploring the relationship between proteins and the risk of AMD, such as the protective role of complement factor H-related protein 1/3 deficiency in AMD (8). However, limitations of observational studies mean that results may be influenced by external variables or reverse causation bias. Mendelian randomization (MR) analysis uses genetic variants as instrumental variables to strengthen causal inference, and compared to observational studies, this method is less susceptible to confounding and reverse causation biases. MR analysis has been widely used to explore associations between plasma proteins and health outcomes. Summary data-based MR (SMR) extends MR analysis and demonstrates greater statistical power when exposure and outcome data can be obtained from two independent samples with large sample sizes (9). Leveraging data from large-scale genome-wide association studies (GWAS) and protein quantitative trait loci (pQTL), we conducted a proteome-wide MR study to investigate the connections between over a thousand plasma proteins and AMD and its related subtypes.

## 2 Materials and methods

### 2.1 Study design

**Figure 1:**
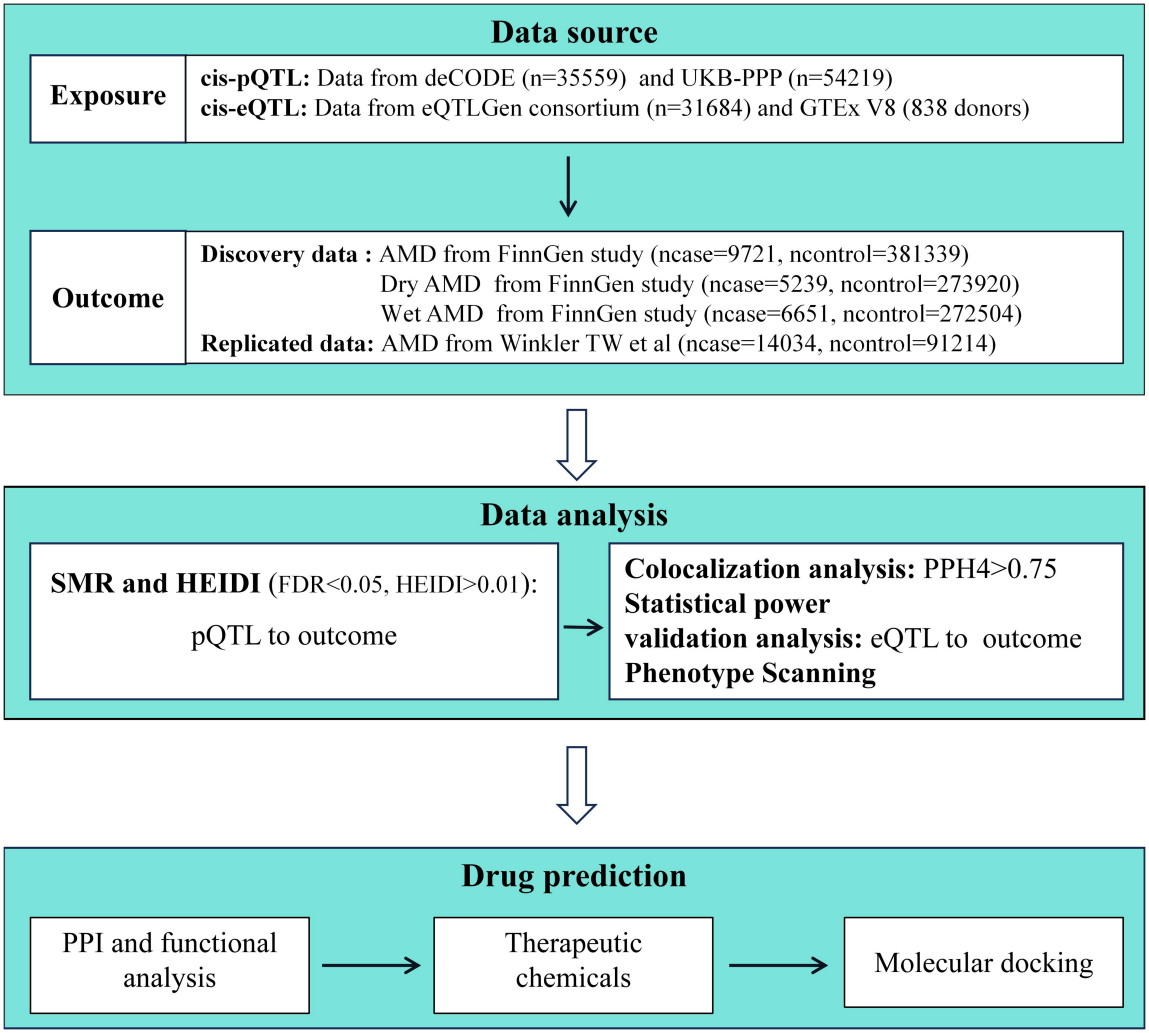
Flowchart of this study.

The study methods were compliant with the STROBE-MR checklist (10), further details can be found in Supplementary Figure S1. First, we applied the SMR method to analyze pQTL and GWAS data. In this study, the exposure variables were two large pQTL datasets, and the outcome variables were GWAS data for AMD and its subtypes. Afterwards, we utilized the Heterogeneity in Dependent Instrument (HEIDI) to test for heterogeneity and conducted power calculations for causal effect estimation using an online power calculator. Subsequently, we further strengthened the causal inference between proteins and AMD through colocalization analysis. For the proteins yielding positive results, we conducted additional validation using data from expression quantitative trait loci (eQTL) in blood samples. To understand the functional characteristics and interactions of the identified target proteins, we constructed a protein–protein interaction (PPI) network. Furthermore, in order to identify potential therapeutic chemical compounds, we searched for protein-related potential chemicals and explored the availability and pharmacological activities of drugs targeting potential AMD targets through molecular docking studies. The specific research workflow is illustrated in Figure 1.

### 2.2 Data sources

The two proteomic datasets were derived from the UK Biobank Pharma Proteomics Project (UKB-PPP) and the deCODE Genetics study in Iceland. The UKB-PPP collected data on 2,923 proteins from plasma samples of 54,219 participants using the Olink platform (11), while the deCODE study analyzed summary-level statistics on genetic associations with plasma protein levels for 4,907 proteins in 35,559 Icelandic individuals (12). Top cis-pQTLs meeting threshold selection (*p* < 5e-8) were chosen as instrumental variables. The blood eQTL data were obtained from the eQTLGen^1^ and the Genotype-Tissue Expression (GTEx) project.^2^ Subsequently, summary data on AMD and its subtypes from the latest release (R10 version) of the FinnGen study were utilized as outcomes. AMD comprised 9,721 cases and 381,339 controls, wet AMD included 5,239 cases and 273,920 controls, and dry AMD included 6,651 cases and 272,504 controls. Detailed information on the data sources is provided in Supplementary Table S1.

### 2.3 Summary-data-based MR analysis

We utilized the SMR method to analyze the correlation between plasma proteins and the risk of AMD and its subtypes, and validated the positive results at the eQTL level. The cis region was defined as the top QTL plus/minus 1,000 kb window, and QTLs within this region that met the specified threshold (*p* < 5e-8) were selected as instrumental variables. Quality control was conducted on allele frequencies, and SNPs were excluded if the allele frequency differences between datasets in pairwise comparisons (including the LD reference sample, the QTL data, and the GWAS summary data) exceeded a specified difference threshold (set at 0.2) (13). HEIDI tests were performed to determine whether there was pleiotropy between the exposure and outcome (9). A *p*-value <0.01 in HEIDI tests indicated potential pleiotropy (14). Additionally, the Benjamini–Hochberg method was utilized to adjust the false discovery rate (FDR) of SMR analysis results to avoid false positives, with FDR < 0.05 considered significant for SMR analysis. Subsequently, we integrated the results of the two protein datasets with the analysis of AMD and its subtypes to broadly screen for targets, retaining deCODE’s SMR analysis data for overlapping proteins. Follow-up colocalization analysis was conducted on proteins that met the criteria of FDR < 0.05 and HEIDI >0.01. SMR analysis and HEIDI tests were performed using SMR software version 1.3.1 and the “DrugTargetMR” package (version 0.2.6) in R software version 4.3.1.

### 2.4 Statistical power

We used an online power calculator to assess the statistical power of the MR analysis to validate the stability of the results^3^ (15). Power values greater than 0.8 were considered to have high statistical power (16).

### 2.5 Colocalization analysis

We performed colocalization analysis to determine whether the selected positive proteins and AMD are driven by the same genetic variants, making the causal relationship between genetic variants and outcomes more authentic and excluding the influence of linkage disequilibrium or other confounding factors (17). The colocalization analysis assumes that causal variables are included in the variable set, and within the genomic region of interest, each trait has at most one association. The colocalization analysis preferably estimates single SNP regression coefficients along with their variances or standard errors, and calculates posterior probabilities (PP) through univariate association *p*-values and MAF values (18). Colocalization analysis results support five hypotheses and report the PP for each hypothesis: H0: the genetic variant is not associated with any trait within the locus; H1: associated with only one trait; H2: associated with another trait; H3: associated with both traits, but does not have a common causal variant; H4: associated with both traits and shares the same causal variant (18). Based on previous studies (19), we set the colocalization window to ±1,000 kb. A posterior probability (PPH4) greater than 0.75 was considered strong evidence for colocalization. The colocalization analysis was conducted using the “DrugTargetMR” package (version 0.2.6) in R software version 4.3.1. The positive proteins identified through SMR and colocalization analysis are considered to be associated with AMD and its subtypes, and further validation was conducted at the blood eQTL level.

### 2.6 Phenotype scanning

To understand whether the identified instrumental variables are closely associated with other traits and exhibit pleiotropy, we conducted a phenotype scan using the PhenoScanner database.^4^ The criteria for phenotype scanning were as follows: (1) GWAS data originated from European populations; (2) the effect allele of the instrumental variable was consistent with our results; (3) the instrumental variable exhibited genome-wide significant correlation (*p* < 5E-8) with the trait.

### 2.7 PPI network, chemical exploration, and molecular docking

To explore potential interactions among the identified proteins in this study, we utilized GeneMANIA^5^ to construct functional and interaction networks of positive proteins for AMD and its subtypes based on SMR and colocalization analysis. We searched for potential therapeutic chemical compounds for the identified proteins through the Comparative Toxicogenomics Database,^6^ which provides information on chemical substance-gene/protein interactions. The most relevant chemical substances to each protein were selected for further analysis. Subsequently, we performed molecular docking of the chemical substances with the corresponding proteins to assess the binding affinity and mode between candidate drug molecules and proteins, identifying high-binding protein-drug interaction patterns. Structural data for the related compounds were obtained from the PubChem Compound database,^7^ while protein structure data were obtained from the Protein Data Bank.^8^ Molecular docking was carried out using AutoDockTools software version 1.5.7, and visualization was completed using pymol software version 2.3.0.

## 3 Results

### 3.1 Results of SMR analysis

In the deCODE dataset, a total of 1733 eligible proteins were subjected to SMR analysis and HEIDI testing for their association with AMD (Supplementary Table S2), Dry-AMD (Supplementary Table S3), and Wet-AMD (Supplementary Table S4). After FDR adjustment (*p* < 0.05) and HEIDI tests (*p* > 0.01), the SMR analysis identified 7, 6, and 8 plasma proteins causally related to AMD, Dry-AMD, and Wet-AMD, respectively. Among them, SFTA2, LTA, BRD2, PILRA, and ACADSB were identified as protective proteins for AMD, while COL10A1 and WARS1 were identified as risk proteins for AMD. The protective effect of SFTA2 was validated in the replicated data for AMD (Supplementary Table S5); SFTA2, LTA, HSPA1L, and ACADSB were identified as protective proteins for Dry-AMD, with COL10A1 and WARS1 as the risk proteins; for Wet-AMD, SFTA2, VARS1, and HSPA1L were identified as protective proteins, while COL10A1, WARS1, VTN, SDF2, and LBP were identified as risk proteins. In the UKB-PPP dataset, a total of 2001 eligible proteins were subjected to SMR analysis and HEIDI testing for their association with AMD (Supplementary Table S6), Dry-AMD (Supplementary Table S7), and Wet-AMD (Supplementary Table S8). After FDR adjustment (*p* < 0.05) and HEIDI tests (*p* > 0.01), the SMR analysis identified 9, 5, and 6 plasma proteins causally related to AMD, Dry-AMD, and Wet-AMD, respectively. Among them, PAXX, TGFB1, and TNFRSF10A were identified as protective proteins for AMD, while IL20RB, ABO, CFD, PILRA, WARS1, and TNFSF14 were identified as risk proteins for AMD. Specifically, the protective effect of TNFRSF10A on AMD was validated in the replicated data (Supplementary Table S9); PAXX and ACADSB were identified as protective proteins for Dry-AMD, while IL20RB, WARS1, and DPEP2 were identified as risk proteins; for Wet-AMD, CD226, TGFB1, and TNFRSF10A were identified as protective proteins, while CSF2, ABO, and WARS1 were identified as risk proteins. WARS1 and PILRA were identified in the SMR analysis of both protein datasets for AMD, with WARS1 showing a consistent risk trend but PILRA showing a different direction, thus PILRA was excluded; for Dry-AMD, the analysis of the two protein datasets for WARS1 and ACADSB showed the same trend; for Wet-AMD, WARS1 showed the same trend in the analysis of both protein datasets. Integrating the SMR analysis results of the two protein datasets, a total of 13, 9, and 13 plasma proteins causally related to AMD, Dry-AMD, and Wet-AMD were identified, and these positive proteins were included in the colocalization analysis (see Table 1 for details).

**Table 1:** Results of SMR and colocalization analysis of plasma proteins with AMD and its subtypes.

Outcomes	Proteins	OR (95%CI)	*p* value after FDR adjustment	*p* value for HEIDI test	PPH4	Power
AMD	SFTA2	0.02 (0.01–0.07)	3.95E-09	0.031	<0.01	1.00
	LTA	0.05 (0.01–0.21)	3.46E-03	0.026	0.07	1.00
	BRD2	0.13 (0.04–0.41)	4.56E-02	0.015	0.79	1.00
	COL10A1	1.47 (1.27–1.71)	7.74E-05	0.233	0.67	0.91
	ACADSB	0.55 (0.40–0.76)	2.59E-02	0.015	<0.01	0.99
	WARS1	1.48 (1.27–1.72)	4.29E-05	0.551	0.99	0.92
	IL20RB	1.75 (1.33–2.32)	3.69E-03	0.086	0.88	0.99
	ABO	1.06 (1.03–1.10)	1.93E-02	0.448	0.11	0.12
	PAXX	0.70 (0.58–0.86)	1.76E-02	0.923	0.01	0.87
	CFD	1.54 (1.22–1.94)	9.88E-03	0.742	0.64	1.00
	TGFB1	0.74 (0.65–0.84)	2.70E-04	0.905	0.99	0.74
	TNFRSF10A	0.88 (0.83–0.94)	2.59E-02	0.155	0.78	0.80
	TNFSF14	1.24 (1.10–1.41)	3.61E-02	0.031	<0.01	0.75
Dry-AMD	SFTA2	0.02 (0.01–0.08)	5.48E-08	0.114	<0.01	1.00
	LTA	0.06 (0.01–0.25)	1.40E-02	0.043	0.07	1.00
	HSPA1L	0.16 (0.10–0.24)	1.67E-13	0.014	<0.01	1.00
	COL10A1	1.46 (1.22–1.75)	3.74E-03	0.112	0.23	0.97
	ACADSB	0.46 (0.31–0.69)	1.28E-02	0.054	<0.01	1.00
	WARS1	1.45 (1.22–1.73)	3.88E-03	0.708	0.95	0.97
	IL20RB	2.07 (1.49–2.89)	9.54E-04	0.072	0.93	1.00
	PAXX	0.67 (0.53–0.84)	2.97E-02	0.734	0.02	0.83
	DPEP2	1.66 (1.28–2.14)	4.84E-02	0.418	<0.01	0.96
Wet-AMD	SFTA2	0.01 (0.00–0.05)	5.41E-08	0.017	<0.01	1.00
	VARS1	0.11 (0.06–0.19)	5.62E-12	0.023	<0.01	1.00
	HSPA1L	0.14 (0.08–0.22)	1.52E-12	0.049	<0.01	1.00
	COL10A1	1.66 (1.36–2.03)	1.37E-04	0.412	0.95	0.95
	WARS1	1.51 (1.24–1.84)	4.43E-03	0.838	0.95	0.85
	VTN	1.06 (1.03–1.09)	3.20E-02	0.332	0.77	0.12
	SDF2	1.37 (1.15–1.63)	3.34E-02	0.172	0.76	0.9
	LBP	1.11 (1.05–1.17)	3.76E-02	0.056	0.75	0.32
	CSF2	1.46 (1.22–1.74)	7.27E-03	0.127	0.95	0.97
	ABO	1.08 (1.03–1.13)	3.61E-02	0.462	0.16	0.2
	CD226	0.75 (0.65–0.88)	1.96E-02	0.231	0.80	0.84
	TGFB1	0.69 (0.58–0.82)	1.82E-03	0.203	0.97	0.97
	TNFRSF10A	0.84 (0.77–0.92)	1.55E-02	0.148	0.87	0.81

### 3.2 Results of colocalization analysis and phenotype scanning

**Figure 2:**
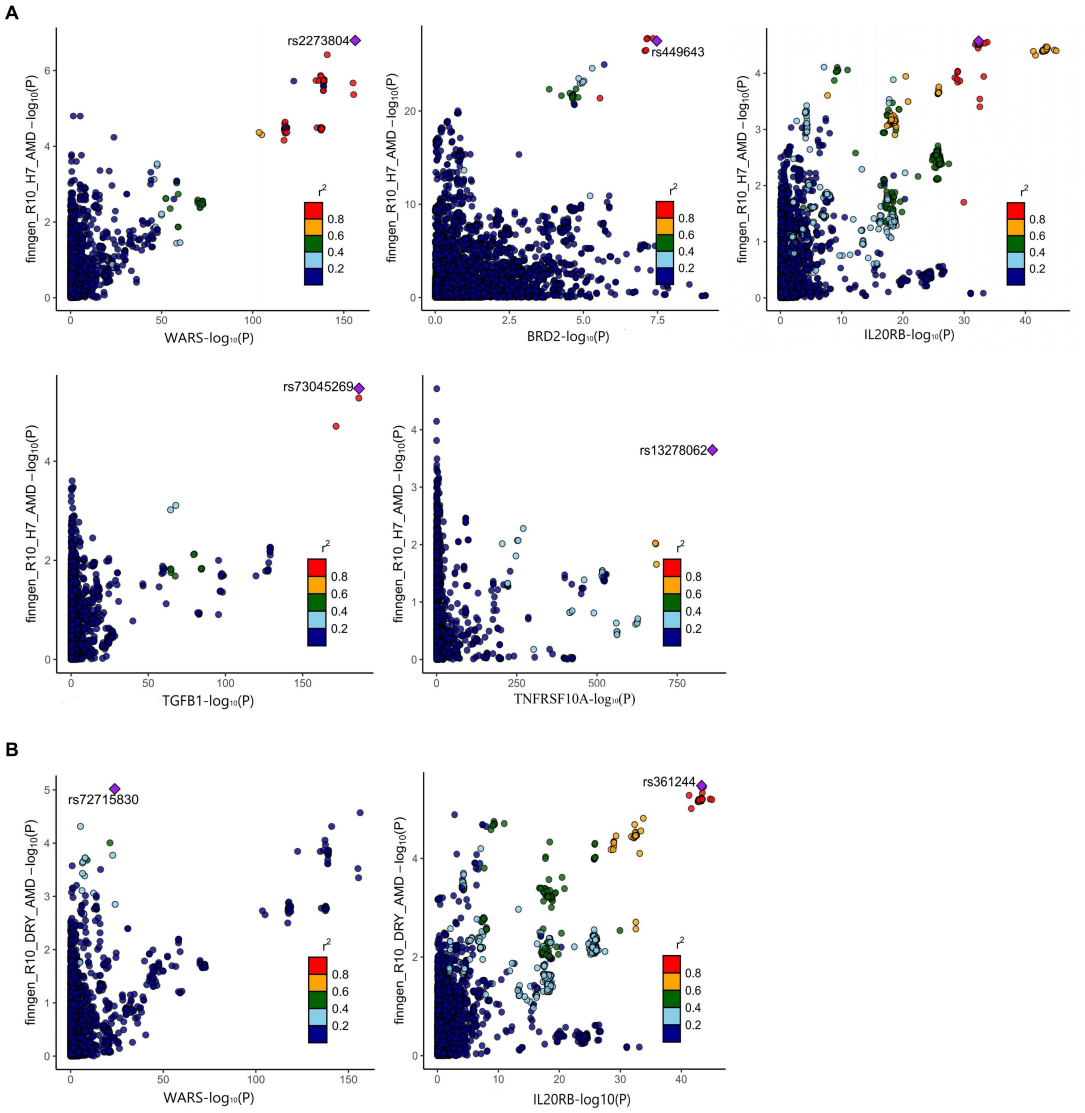
Result of colocalization analysis. **(A)** Colocalization analysis for AMD; **(B)** Colocalization analysis for DRY-AMD.

**Figure 3:**
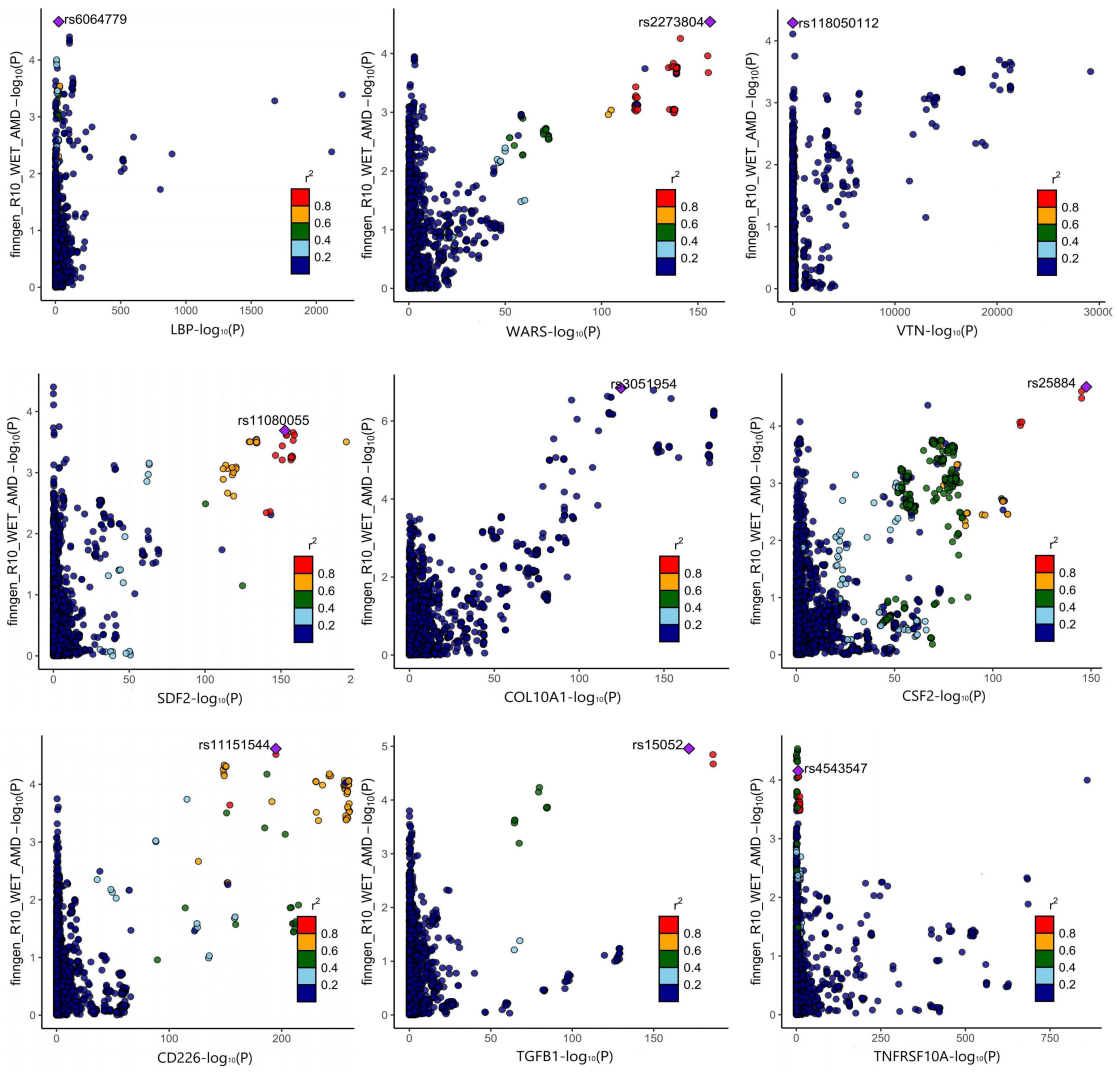
Result of colocalization analysis for WET-AMD.

The positive proteins identified in the SMR analysis underwent co-localization analysis with AMD (Supplementary Tables S10, S13), Dry-AMD (Supplementary Tables S11, S13), and Wet-AMD (Supplementary Tables S12, S15). The findings reveal evidence of co-localization between five proteins (WARS1, BRD2, IL20RB, TGFB1, TNFRSF10A) and AMD (Figure 2A). Furthermore, two proteins (WARS1, IL20RB) exhibit co-localization support with Dry-AMD (Figure 2B). Additionally, nine proteins are associated with co-localization in Wet-AMD (Figure 3), sharing causal variation, including COL10A1, WARS1, VTN, SDF2, LBP, CD226, TGFB1, TNFRSF10A, CSF2. Refer to Table 1 for specifics.

The phenotype scan results revealed associations between BRD2 and traits such as lymphocyte count, oropharynx cancer, and nuclear pore membrane glycoprotein 210-like levels. IL20RB showed associations with phenotypes including prostate cancer, monocyte count, and carpal tunnel syndrome. COL10A1 exhibited associations with systemic lupus erythematosus and liver enzyme levels. Please refer to Supplementary Table S16 for details. There is no evidence indicating a direct association between these phenotypes and osteoarthritis.

### 3.3 Validation analysis

To validate our findings at the gene expression level, we conducted additional validation analyses using blood eQTL data for the positive proteins identified through SMR and colocalization analyses. We excluded VTN, SDF2, LBP, CSF2, and TGFB1 due to the absence of qualifying eQTLs in both datasets. The final results are presented in Table 2. In the eQTLGen dataset, increased expression of IL20RB was associated with higher risk of AMD (OR = 2.09, 95%CI: 1.74–2.84, *p* = 1.61E-02) and Dry AMD (OR = 2.31, 95%CI: 1.14–4.66, *p* = 2.01E-02), while expression of WARS1 was associated with decreased risk of AMD (OR = 0.90, 95%CI: 0.86–0.95, *p* = 4.42E-05), Dry AMD (OR = 0.91, 95%CI: 0.85–0.96, *p* = 1.94E-03), and Wet AMD (OR = 0.89, 95%CI: 0.83–0.95, *p* = 9.06E-04). TNFRSF10A expression was associated with reduced risk of AMD (OR = 0.84, 95%CI: 0.74–0.95, *p* = 8.72E-03) and Wet AMD (OR = 0.79, 95%CI: 0.67–0.93, *p* = 5.73E-03), while CD226 expression was associated with decreased risk of Wet AMD (OR = 0.79, 95%CI: 0.71–0.89, *p* = 9.36E-05). These results passed the HEIDI test, but only the effects of WARS1 on AMD and CD226 on Wet AMD remained significant after FDR correction (P_FDR_ = 4.46E-03 and P_FDR_ = 1.04E-02). In the GTEx dataset, expression of WARS1 was associated with reduced risk of AMD (OR = 0.87, 95%CI: 0.81–0.93, *p* = 4.96E-05), Dry AMD (OR = 0.88, 95%CI: 0.82–0.95, *p* = 1.87E-03), and Wet AMD (OR = 0.86, 95%CI: 0.79–0.94, *p* = 1.07E-03), while TNFRSF10A expression was associated with decreased risk of AMD (OR = 0.81, 95%CI: 0.71–0.91, *p* = 5.22E-04) and Wet AMD (OR = 0.73, 95%CI: 0.62–0.87, *p* = 2.79E-04). These associations also passed the HEIDI test, but only the protective effect of WARS1 on AMD remained significant after FDR correction (P_FDR_ = 1.47E-02). The protective effect of WARS1 expression on AMD was validated in both datasets.

**Table 2:** The eQTL data of positive results and SMR analysis of GWAS.

Outcomes	Proteins	Origins	OR (95%CI)	*P* value	*P* value after FDR adjustment	*P* value for HEIDI test
AMD	BRD2	eqtlGen	1.18 (0.93–1.34)	2.31E-01	7.53E-01	0.009
		GTEx	-	-	-	-
	WARS1	eqtlGen	0.90 (0.86–0.95)	4.42E-05	4.46E-03	0.070
		GTEx	0.87 (0.81–0.93)	4.96E-05	1.47E-02	0.307
	IL20RB	eqtlGen	2.09 (1.47–3.84)	1.61E-02	2.89E-01	0.414
		GTEx	-	-	-	-
	TNFRSF10A	eqtlGen	0.84 (0.74–0.95)	8.72E-03	2.10E-01	0.486
		GTEx	0.81 (0.71–0.91)	5.22E-04	1.17E-01	0.131
Dry-AMD	WARS1	eqtlGen	0.91 (0.85–0.96)	1.94E-03	1.07E-01	0.171
		GTEx	0.88 (0.82–0.95)	1.87E-03	2.38E-01	0.503
	IL20RB	eqtlGen	2.31 (1.14–4.66)	2.01E-02	4.06E-01	0.887
		GTEx	-	-	-	-
Wet-AMD	COL10A1	eqtlGen	0.89 (0.61–1.33)	5.96E-01	9.36E-01	0.202
		GTEx	-	-	-	-
	WARS1	eqtlGen	0.89 (0.83–0.95)	9.06E-04	7.42E-02	0.384
		GTEx	0.86 (0.79–0.94)	1.07E-03	2.23E-01	0.644
	CD226	eqtlGen	0.79 (0.71–0.89)	9.36E-05	1.04E-02	0.488
		GTEx	-	-	-	-
	TNFRSF10A	eqtlGen	0.79 (0.67–0.93)	5.73E-03	2.45E-01	0.415
		GTEx	0.73 (0.62–0.87)	2.79E-04	1.06E-01	0.154

### 3.4 Results of PPI, chemical exploration, and molecular docking

**Figure 4:**
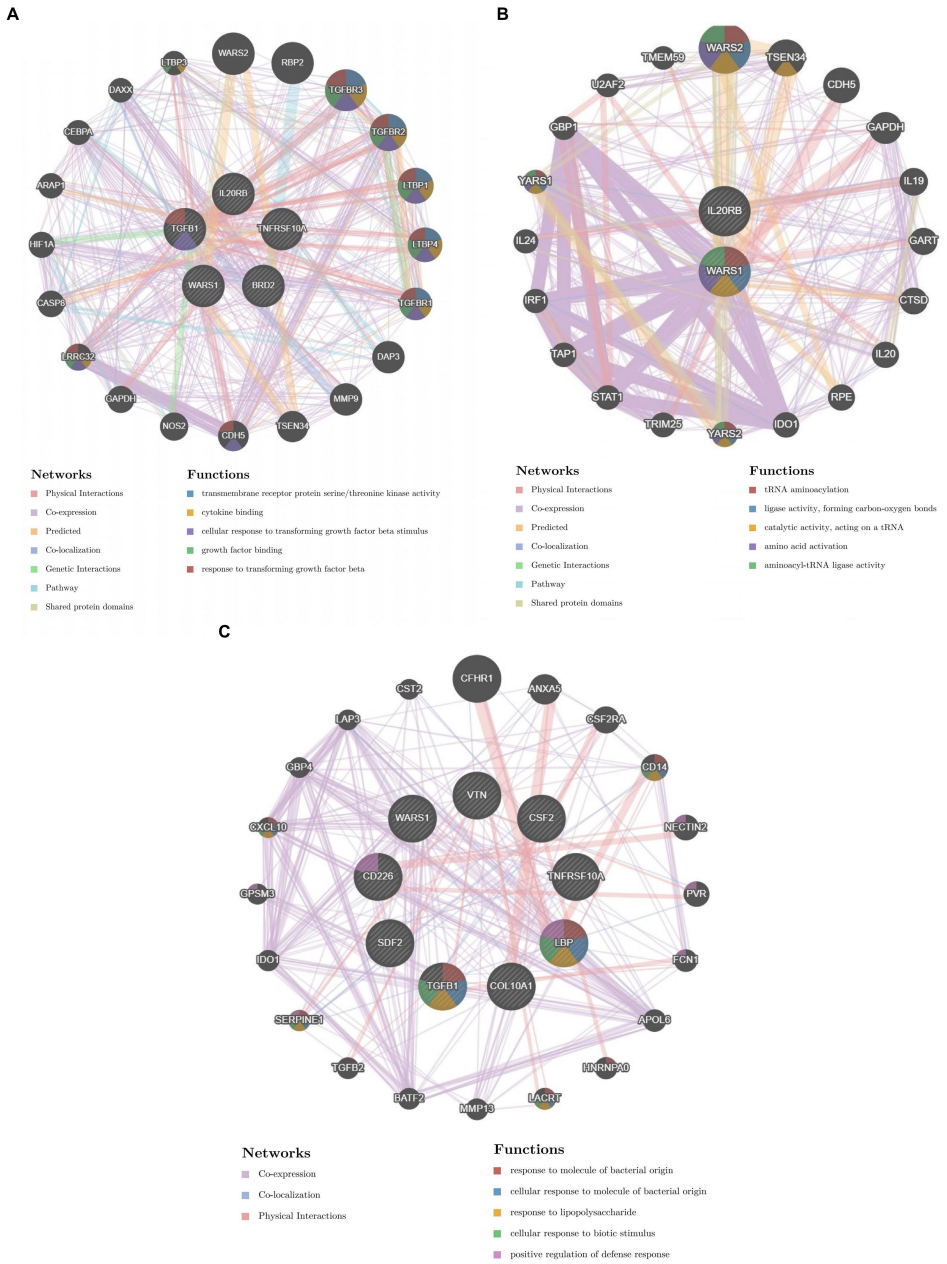
PPI results.

**Figure 5:**
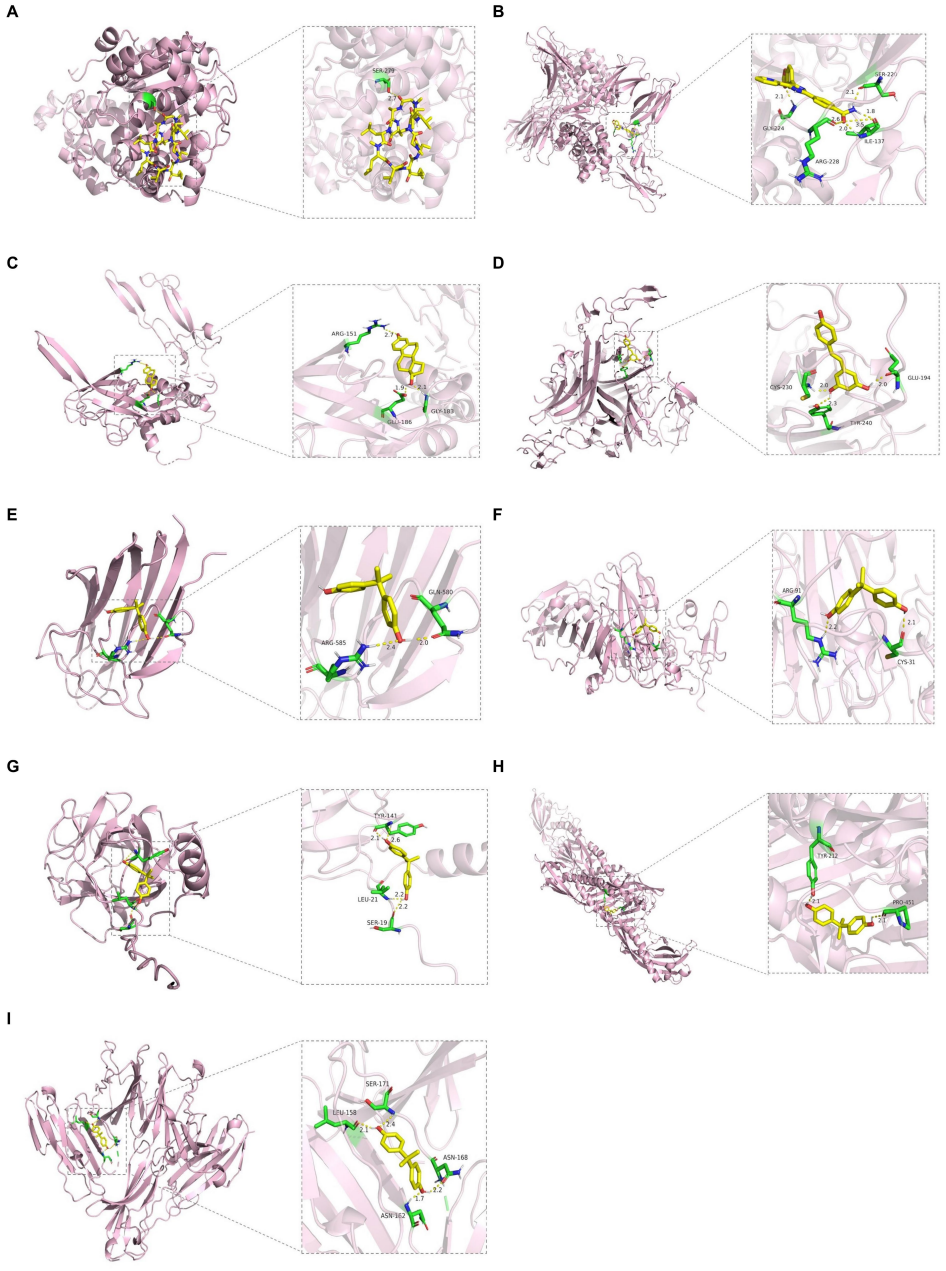
Molecular docking results. **(A)** WARS1 docking cyclosporine. **(B)** IL20RB docking 4-(5-benzo(1,3)dioxol-5-yl-4-pyridin-2-yl-1H-imidazol-2-yl) benzamide. **(C)** TGFB1 docking estradiol. **(D)** TNFRSF10A docking resveratrol. **(E)** COL10A1 docking bisphenol A. **(F)** VTN docking bisphenol A. **(G)** SDF2 docking bisphenol A. **(H)** LBP docking bisphenol A. **(I)** CD226 docking bisphenol A.

We constructed the PPI networks of the positive proteins for AMD and its subtypes using GeneMANIA. As shown in Figure 4, in addition to interacting with each other, they also interact with around 20 surrounding potential proteins, generating hundreds of interaction links. In the PPI network of AMD-related proteins, these connections mainly include Physical Interactions (70.9%), Co-expression (16.01%), Predicted (4.96%), etc. For Dry-AMD and Wet-AMD, the connections mainly include Physical Interactions, Co-expression, Co-localization, etc. Furthermore, the top five significantly enriched functional pathways were analyzed, showing network functions such as transmembrane receptor protein serine/threonine kinase activity, tRNA aminoacylation, response to molecule of bacterial origin, etc. Potential therapeutic chemicals were searched using the Comparative Toxicogenomics Database, and we selected chemicals most relevant to the corresponding proteins. Detailed information can be found in Table 2. Candidate chemical structures and protein structures were obtained from PubChem and PDB databases for molecular docking to evaluate the affinity between the chemicals and their target proteins. Due to the absence of some molecular structures, molecular docking was performed for 9 protein-molecule pairs to generate corresponding binding energies. The binding energies of most protein-molecule pairs were low, indicating stable binding through visible hydrogen bonds and strong electrostatic interactions with their protein targets, with each ligand successfully occupying the binding pocket of the candidate molecules. Among them, IL20RB and 4-(5-benzo(1,3)dioxol-5-yl-4-pyridin-2-yl-1H-imidazol-2-yl) benzamide showed the lowest binding energies, indicating highly stable binding. For specific details, please refer to Tables 2, 3 and Figure 5.

**Table 3:** Chemical exploration of identified proteins and their molecular docking results.

Outcomes	Target	PDB ID	Chemicals	PubChem CID	Binding energy
AMD	WARS1	1O5T	Cyclosporine	5284373	−3.07
	BRD2	1X0J	lipopolysaccharide, *Escherichia coli* O111 B4	-	-
	IL20RB	4DOH	4-(5-benzo(1,3)dioxol-5-yl-4-pyridin-2-yl-1H-imidazol-2-yl)benzamide	4521392	−8.58
	TGFB1	5VQP	Estradiol	5757	−6.01
	TNFRSF10A	5CIR	Resveratrol	445154	−6.70
Dry-AMD	WARS1	1O5T	Cyclosporine	5284373	−3.07
	IL20RB	4DOH	4-(5-benzo(1,3)dioxol-5-yl-4-pyridin-2-yl-1H-imidazol-2-yl)benzamide	4521392	−8.58
Wet-AMD	COL10A1	1GR3	Bisphenol A	6623	−5.46
	WARS1	1O5T	Cyclosporine	5284373	−3.07
	VTN	3BT1	Bisphenol A	6623	−5.77
	SDF2	SDF2	Bisphenol A	6623	−5.37
	LBP	4M4D	Bisphenol A	6623	−5.61
	CD226	6ISB	Bisphenol A	6623	−6.36
	TGFB1	5VQP	Estradiol	5757	−6.01
	TNFRSF10A	5CIR	Resveratrol	445154	−6.70
	CSF2	2GMF	Lipopolysaccharides	-	-

## 4 Discussion

This study identified 5 proteins (WARS1, BRD2, IL20RB, TGFB1, TNFRSF10A) associated with AMD through SMR and colocalization analysis, 2 proteins (WARS1, IL20RB) associated with Dry-AMD proteins, and 9 proteins (COL10A1, WARS1, VTN, SDF2, LBP, CD226, TGFB1, TNFRSF10A, CSF2) associated with Wet-AMD. Subsequently, the biological interaction relationships of these proteins were elucidated using the PPI network. Potential therapeutic chemicals for these proteins were then explored, and molecular docking of proteins with the interacting chemicals was performed, demonstrating their therapeutic value.

AMD is the result of multifactorial interactions, with inflammation believed to play a significant role in the pathogenesis of AMD. Local inflammation leads to degeneration of the retinal pigment epithelium (RPE), Bruch’s membrane damage, and the development of choroidal neovascularization (20, 21). Colony stimulating factor 2 (CSF2) is a hematopoietic growth factor that primarily acts to stimulate the formation of colonies of bone marrow cells to produce granulocytes and macrophages (22). CSF2 affects various bone marrow cell lines, including macrophages and neutrophils, by inducing these cells to produce cytokines involved in the inflammatory response (23). This highlights the important biological role of CSF2 in regulating bone marrow cell development and inflammation modulation. Previous studies have shown that CSF2 is expressed in astrocytes in the central nervous system through stimulation by IL-1β (24). Additionally, research has indicated that CSF2 can induce proliferation of microglia in hippocampal slice cultures without inducing the production of pro-inflammatory cytokines (25). Recently, Kosuke Saita et al. (26) explored the role of CSF2-induced microglia in modulating retinal inflammation in retinal degeneration. The study found that CSF2 was strongly induced in the retina and led to upregulation of C-C motif chemokine ligand 2 (Ccl2) and C-X-C motif chemokine ligand 10 (Cxcl10) in activated microglia, indicating that CSF2 triggers a robust inflammatory response in the retina. We predicted the potential action of lipopolysaccharide (LPS) on CSF2. LPS is a major cell wall component of Gram-negative bacteria, and numerous studies have shown that LPS induces increased expression of CSF2 protein, such as the expression of GM-CSF at mRNA and protein levels in LPS-induced MDA-MB-231 cells (27). In AMD, LPS induces inflammatory responses in retinal pigment epithelium, increasing the risk of AMD (28). Our results suggest that increased CSF2 protein expression may elevate the risk of AMD, and our hypothesis is that LPS-induced stimulation of CSF2 protein expression exacerbates retinal inflammation leading to AMD, which requires further validation.

Bromodomain-containing proteins (BRDs) are substances involved in protein–protein interactions, serving as recruiting platforms that link protein complexes with acetylated histones (29, 30). Dysregulation of proteins containing bromodomains leads to changes in acetylation levels, thereby promoting abnormal expression of inflammatory cytokines and causing inflammation. Bromodomain extra-terminal (BET) proteins, such as BRD2, are important members of the bromodomain-containing protein family. Previous studies have shown that BET proteins promote gene transcription in inflammation by recruiting the transcriptional coactivator P-TEFb (31). Therefore, BET proteins may serve as valuable targets for treating inflammatory diseases.

Transforming growth factor beta 1 (TGFB1) is an important cytokine that has been shown to promote fibrosis and inflammatory responses, as well as regulate proliferation, differentiation, apoptosis, adhesion, and migration of various cell types (32). In addition, TGFB1 can induce the synthesis of extracellular matrix (ECM) proteins, participate in angiogenesis, endothelial cell proliferation, ECM deposition, and disruption of the blood-retinal barrier (33, 34). TGFB1 plays a significant role in the process of angiogenesis (35, 36). Previous studies have indicated that TGFB1 can restrict angiogenic potential by acting on CLENDO cells (37). We predicted the chemical compound Estradiol as a potential agent acting on TGFB1, and protein docking studies also revealed stable binding affinity. Animal experiments have shown that 17β-estradiol (E2) can inhibit the expression of TGFB1 mRNA in normal mouse pituitary prolactin cells (38). E2 reduces TGFB1 protein activity, protecting foot cells from TGFB1-induced apoptosis (39). Research by M. Pastorcic et al. suggests that estradiol-17 beta intervention during pituitary tumor development reduces TGFB1 protein levels in anterior pituitary tissue (40). Additionally, TGFB1 is one of the main targets of estrogen stimulation, and Estradiol has a significant impact on vascular endothelial growth factor (VEGF) signaling, aiding in angiogenesis (41). While studies on their role in retinal-related tissues have not been found, based on existing research, we speculate that Estradiol may influence retinal neovascularization by inhibiting TGFB1 protein activity and expression, potentially playing a role in AMD. However, further research is needed to elucidate the specific clinical mechanisms.

TNF Receptor Superfamily Member 10a (TNFRSF10A) is the receptor for the cytokine TNF-related apoptosis-inducing ligand (TRAIL), and it is involved in apoptosis, necrosis, and inflammatory signaling pathways (42, 43). TRAIL binds to TNFRSF10A, and TNFRSF10A initiates the receptor pathway by exposing its cytoplasmic death domain (44). Dysfunction of retinal pigment epithelium (RPE) is one of the pathological changes in AMD. Tumor necrosis factor receptor superfamily member 10A (TNFRSF10A)-LOC389641 shares the same SNP (rs13278062), which has been identified to be associated with AMD risk in previous genome-wide association studies (45, 46). Based on this, Kenichiro Mori et al. elucidated that downregulation of TNFRSF10A expression leads to inactivation of protein kinase C-alpha (PKCA) signaling and results in cellular vulnerability of RPE cells through studies using RPE cells and TNFRSF10A knockout mice (47). This may be the reason why TNFRSF10A plays a protective role in AMD. We hypothesized that the chemical compound Resveratrol may act on TNFSFR10A. It belongs to the stilbenoid family, is a polyphenolic plant toxin, and has been shown to prevent apoptosis in human RPE cells *in vitro* (48). Resveratrol protects or delays H2O2-induced RPE cell death through its antioxidant properties and exhibits potent anti-inflammatory characteristics, significantly inhibiting CXCL11 induction by pro-inflammatory cytokines, thus exerting a protective effect against AMD (49, 50). However, the pathway through which Resveratrol stimulates TNFSFR10A protein in AMD remains to be further elucidated, and our findings provide direction for this.

Vitronectin (VTN) protein is a plasma protein widely present in the human circulatory system, distributed in the extracellular matrix (ECM) of various tissues, including the retina. Specifically, it can be detected in ocular tissues such as the choroid, Bruch’s membrane, and RPE (51, 52). Vitronectin interacts with multiple ligands, participating in processes such as cell adhesion and migration, immune responses, angiogenesis, and fibrinolysis (53–56). Accumulation of sub-RPE deposits is one of the pathological hallmarks of AMD. Several studies have identified vitronectin as a major component and coordinating factor in the formation of AMD-related retinal deposits (57–59). In this study, Vitronectin also showed a trend toward increased risk for AMD. Additionally, research indicates that Vitronectin levels in tissues increase with the presence of inflammation and age (60–62), with age being a significant risk factor for AMD, which aligns with the role of Vitronectin in AMD development. We hypothesized that the chemical compound bisphenol A (BPA) may act on VTN, as it is an estrogenic compound. Current research suggests that BPA induces apoptosis in ARPE-19 cells through downregulation of the Nrf2/HO-1 pathway under oxidative stress and mitochondria-dependent apoptotic pathways (63). Although some studies have reported an increase in VTN protein expression with BPA (64) and docking studies have shown stable binding affinity, the specific mechanism by which BPA acts through the VTN pathway in AMD remains unclear.

The strength of this study lies in the utilization of MR and colocalization methods to jointly estimate the causal effects of plasma proteins on AMD and its subtypes through genetic variations. Additionally, we integrated the results from two protein datasets to comprehensively screen feasible therapeutic targets for AMD. There are several notable limitations to this study. Firstly, the study population being of European descent restricts the generalizability of the results to other populations. Secondly, although colocalization analysis reduces biases that may arise due to linkage disequilibrium, horizontal pleiotropy may not necessarily be minimized. Uncertainty in attribution exists if the variance of regression coefficients is estimated through minor allele frequencies of genotyped SNPs and sample sizes. Additionally, interpretation of the posterior probability of colocalization (PPH4) should be cautious, as smaller outcomes of PPH4 may not represent evidence against joint localization, especially when PPH3 is also low (18). Furthermore, molecular docking can only predict the binding state of drug molecules and proteins, without reflecting the metabolism and pharmacological effects of drug molecules *in vivo*. Therefore, molecular docking can only serve as a supplementary and guiding tool. Finally, the biological mechanisms of targeting proteins and related chemical substances require further *in vitro* and *in vivo* experimental results to elucidate, in order to better understand the therapeutic effects of targeting proteins.

## 5 Conclusion

In conclusion, this study identified potential protein targets for AMD and its subtypes through SMR and colocalization analysis, expanding the current biomarkers for AMD and its subtypes. The study also searched for and validated actionable chemical compounds for the identified proteins. It is hoped that our research findings will contribute to the development of targeted drugs for AMD and its subtypes.

## Data availability statement

The original contributions presented in the study are included in the article/Supplementary material, further inquiries can be directed to the corresponding author.

## Ethics statement

Ethical review and approval was not required for the study on human participants in accordance with the local legislation and institutional requirements. Written informed consent from the patients/participants or patients/participants' legal guardian/next of kin was not required to participate in this study in accordance with the national legislation and the institutional requirements.

## Author contributions

K-LP: Formal analysis, Supervision, Writing – original draft. HK: Data curation, Formal analysis, Writing – original draft. LL: Conceptualization, Supervision, Writing – review & editing.

## Footnotes

^1^https://www.eqtlgen.org/

^2^https://www.genome.gov/Funded-Programs-Projects/Genotype-Tissue-Expression-Project

^3^https://sb452.shinyapps.io/power/

^4^http://www.phenoscanner.medschl.cam.ac.uk/

^5^http://www.GeneMANIA.org

^6^https://ctdbase.org/

^7^https://pubchem.ncbi.nlm.nih.gov/

^8^https://www.rcsb.org/
